# Title page: psychometric properties of literacy of suicide scale (LOSS) in iranian population: long form

**DOI:** 10.1186/s12889-023-15528-8

**Published:** 2023-03-30

**Authors:** Alireza Jafari, Mahdi Moshki, Ali Mohammad Mokhtari, Amirarsalan Ghaffari, Mahbobeh Nejatian

**Affiliations:** 1 Department of Health Education and Health Promotion, School of Health, Social Development and Health Promotion Research Center, Gonabad University of Medical Sciences, Gonabad, Iran; 2 Department of Health Education and Health Promotion, School of Health, Social Determinants of Health Research Center, Gonabad University of Medical Sciences, Gonabad, Iran; 3 Department of Epidemiology and Biostatistics, School of Health, Social Development and Health Promotion Research Center, Gonabad University of Medical Sciences, Gonabad, Iran; 4 Student Research Committee, Gonabad University of Medical Sciences, Gonabad, Iran; 5 Social Determinants of Health Research Center, Gonabad University of Medical Sciences, Gonabad, Iran

**Keywords:** Validity, LOSS-26, Reliability, Literacy of suicide, Health literacy suicide, Public population

## Abstract

**Introduction:**

Suicide and suicide attempts are among the most important indicators of mental health in the world. In this research, the validity and reliability of Literacy of Suicide Scale (LOSS) was examined in general people over the age of 18.

**Methods:**

This cross-sectional psychometric study was conducted in 2022 among 952 general population in Iran. Participants were selected by two methods of proportional stratified sampling and simple random sampling. The internal consistency of the tools was assessed using Cronbach’s alpha coefficient, and McDonald omega coefficient. Also, test-retest reliability was checked by Intraclass Correlation Coefficient (ICC).

**Results:**

In the confirmatory factor analysis section, the factor loading of all questions were above 0.4 and one questions were deleted and final model with four factors and 25 questions was confirmed (Some of goodness-of-fit indexes: AGFI = 0.910, RMSEA = 0.050, IFI = 0.901, and χ2/df = 3.333). For all questions, the Cronbach’s alpha coefficient was 0.859, McDonald omega coefficient was 0.866, and ICC was 0.895. Finally, the Persian long version of LOSS was approved with 25 items and four subscales: causes/triggers (9 items), risk factors (7 items), signs and symptoms (5 items) and treatment/prevention (4 items).

**Conclusion:**

The Persian long version of LOSS with four subscales and 25 items is an appropriate tool to investigate the state of suicide literacy in the public population.

**Supplementary Information:**

The online version contains supplementary material available at 10.1186/s12889-023-15528-8.

## Introduction

Suicide is a conscious act that leads to death [1]. Suicide and suicide attempt are an important issue of public concern. In other words, suicide and suicide attempt are among the most important indicators of mental health for individuals in society [2]. The number of suicides has been on the rise over the past 50 years, and in many countries actual suicide rates are not published due to cultural and ethnic concerns [3].

According to the results of the 2020 systematic review, the prevalence of suicide beliefs in the general population was 12.9%, with 15.8% among women and 5.2% among men. Also, the prevalence of suicide was 8.8% after a disaster [4]. The results of another systematic review in 2020 showed that the prevalence of suicide beliefs ranged from 9.7 to 58.3%, and the suicide rate ranged from 0.7 to 14.7% [5].

The results of a study in Iran showed that suicide rates are high, with most suicides occurring between the ages of 20 and 29 [6]. Health literacy is one of the variables that affects people’s behavior [7]. High health literacy has many benefits such as prevention, early diagnosis and intervention in the early stages, and reduced symptoms related to the disease [8]. Suicide literacy is part of mental health literacy and is defined as an understanding of warning signs/ symptoms, causes/ triggers, risk factors, and treatment/prevention [9]. About 80% of those who commit suicide showed signs and symptoms before acting. These results demonstrate the need for suicide literacy in the community and suggest that increasing suicide literacy in the community can help prevent these behaviors [10]. These symptoms are associated with talks about suicide, has a problem in eating or sleeping, experience severe changes in behavior, exit from social activities or friends, gives you its valuable assets, have already committed suicide, takes unnecessary risks, busy with the thought of death and dying, increases the use of alcohol or drugs, loses his interest in his personal appearance [10].

Low literacy in suicide refers to limited public knowledge about suicide, and this low level of literacy can affect people with suicide thoughts or behaviors [11]. Scientific evidence suggests that individuals with misconceptions about risk factors, treatments, and symptoms of suicide behavior may be at risk for suicide thoughts or behavior [11]. Low awareness of suicide also hinders access to specialized mental health services [12]. Another study showed a positive significant correlation between suicide literacy and seeking psychological help, and increased levels of suicide literacy can increase help seeking [13]. The results in studies in different countries have shown that the rate of suicide literacy is low [14–17].

Appropriate tools are needed in order to examine individual conditions in different domains and to design and implement appropriate educational and interventional programs [18]. In Iran, no instrument was observed to examine the state of suicide literacy. One of the best tools for examining suicide literacy is a questionnaire designed by Calear et al. The questionnaire contains 26 questions that evaluate people’s knowledge of suicide and in four areas, including signs/ symptoms of suicide, causes/ triggers, risk factors, treatment and prevention [19]. This tool has been evaluated in several studies in different countries [13, 16, 20–23]. Due to the lack of appropriate suicide literacy tools in Iranian community and the need for availability of this scale, this psychometric study was conducted among general population in Iran.

## Methods

This cross-sectional study assessed the psychometric properties of long version of LOSS among 954 Iranian public participants in Gonabad city in 2022.

### Sample size

In factor analysis, sample size of 100, 200, 300, 500, 1000 and more are consider poor, fair, good, very well, and excellent, respectively [24, 25]. In the CFA section, the sample size of 954 participants was chosen to assess CFA.

### Sampling

In this study, two methods of proportional stratified sampling and simple random sampling were used for the selection of participants. At first, the number of health care centers and their population in Gonabad city was identified. Any healthcare center is then considered a stratum, and within each stratum, participants are selected by simple random sampling. Inclusion criteria of age over 18 years, no cognitive problems, residency in Gonabad city for more than one year and informed consent were considered in selecting the participants.

### Instruments

1. **Demographic section**: In this section, issues such as marital status, age, occupation, sex, and education level were examined.
2. **Literacy of Suicide Scale (LOSS-26)**: The long version of LOSS was designed and assessed by Calear et al. This scale consists of 26 questions that survey the people’s knowledge of suicide and in four dimensions of signs and symptoms (5 items), causes/ triggers (10 items), risk factors (7 items), and treatment and prevention (4 items) [26]. The questions are measured as “true”, “I don’t know” and “false”. Each question has a correct answer and each answer is awarded one point. To the each “wrong answer” and “I do not know answer” the zero score and to each “correct answer” one score are assigned. In view of the fact that 1 question was finally deleted in this study, the questionnaire was determined to be 25 questions, and the item scores ranged from 0 to 25, with high scores indicating good suicide literacy.

### Translation/ cultural adaptation section

This part was checked by WHO Guideline [27]. Before translated the tool, from the designer of the questionnaire, got permission. First, the original English version of LOSS was translated into Persian by two psychologists and health education and health promotion, and the two translated scale were compared and a single Persian version of the LOSS was created. In the next step, the Persian version of the LOSS was translated into English by two experts and then compared with the original English version of the LOSS. After that, the English version was translated into Persian and the final Persian version of LOSS was created.

#### Validity

Based on the source, quantify of content validity and quantify of face validity are not required for standard questionnaires [28]. Because in this study, the LOSS is a standard questionnaire, only quality method was used for evaluation the face and content validity. To investigate the quality content face method, the final version of Persian was examined in terms of the desirability of the expressions in terms of clarity (use of simple and understandable words), the use of a common language (avoiding technical and specialized words). To investigate the quality content validity method, the questionnaire was examined by the specialists in terms of grammar compliance, the use of appropriate words, the importance of items, the placement of items in their proper place, the time to complete the designed tool.

##### CFA

The software of AMOS V.24 was used to evaluate the CFA. Before the running the CFA, the Mahalanobis test was used to determine the outlier’s data and eliminate inappropriate data. Also, kurtosis and skewness tests were used to check the normality of the data. The final model was assessed by using the goodness of fit indexes of RMSEA (root mean square error of approximation), IFI (incremental fit index), PCFI (parsimony comparative fit index), GFI (goodness of fit index), PGFI (parsimony goodness of fit index), CFI (comparative fit index), χ2/df (chi-square ratio to degree of freedom), AGFI (adjusted goodness of fit index), and PNFI (parsimonious normed fit index) [29–31]. Standard indexes to confirm the final model are RMSEA less than 0.08, χ2/df less than 5, PNFI, PGFI, and PCFI more than 0.5, CFI, IFI, and GFI more than 0.9, and AGFI more than 0.8 [29–32].

### Reliability

SPSS software version 20 was used to survey the internal consistency (Cronbach’s alpha coefficient). For internal reliability, a range score between 0.70 and 0.95 is acceptable [33, 34]. The McDonald’s omega coefficient was checking by using JASP (Version ._0.11.1_). In this study, 30 participants were selected to assess test-retest reliability (twice, over a one-month period). To check test-retest reliability, the Intraclass correlation coefficient (ICC) was used, and ICC > 0.80 is acceptable [35]. For calculation the ICC, the model of Two- Way Mixed and type of Absolute Agreement were used.

## Results

### Demographic characteristics

The mean (± standard deviation) age of people was 33.35 (± 12.96). Most of people were married (n = 532, 55.9%) and female (n = 501, 88.9%). The occupational status of most people were university students (n = 375, 39.4%), employed (n = 250, 26.3%), and self-employed (n = 136, 14.3%), respectively. The educational level of the majority was a bachelor’s degree (n = 350, 36.8%) and a diploma (n = 265, 27.8%). Additional demographic information is included in Table 1.

**Table 1:** Frequency distribution of demographic characteristics (n = 952)

Variables		N	%
**Sex**	Male	451	11.1
	Female	501	88.9
**Marital status**	Married	532	55.9
	Single	397	14.7
	Divorced	23	2.4
**Occupation**	Housewife	103	10.8
	University student	375	39.4
	Employed	250	26.3
	Retired	48	5
	Self-employed	136	14.3
	laborer	22	2.3
	Unemployed	18	1.9
**Education level**	Illiterate	2	0.2
	Elementary school	17	1.8
	Middle school	25	2.6
	High school	35	3.7
	Diploma	265	27.8
	Associate degree	136	14.3
	Bachelor degree	350	36.8
	Master’s degree or high degree	122	12.8

### Validity assessment

Qualitative face validity and content validity were evaluated by 8 exerts (Psychologist and health education and promotion) and also by some participants. In this section, 5 questions were modified.

## CFA

**Fig. 1:**
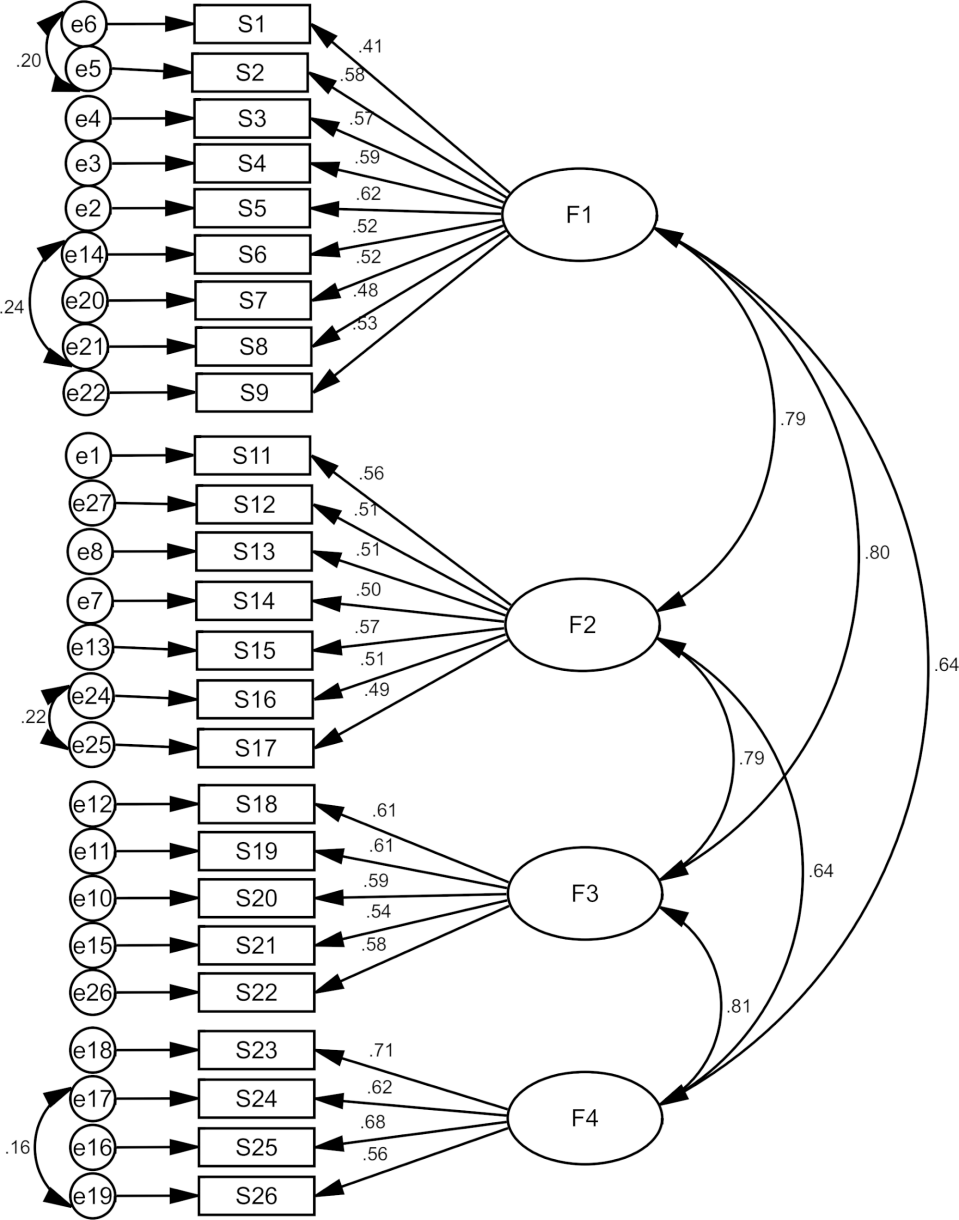
Standardized parameter estimates for the factor structure of the of long form of suicide literacy (F1: Causes/triggers, F2: Risk factors, F3: Signs/ symptoms, F4: Treatment/Prevention)

Based on the results, all goodness-of-fit indexes were acceptable (for example: χ2/df = 3.333, RMSEA = 0.050, IFI = 0.901, AGFI = 0.910) (Table 2). In this section, the factor loading of all questions were above 0.4 and only one question (S10: A person who suicides is mentally ill) was deleted and final model with four factors and 25 questions was confirmed (Table 3; Fig. 1, Table S1).

**Table 2:** The model fit indicators of the Persian version of long form of LOSS

Goodness of fit indices	Confirmatory factor analysis	Acceptable value
**χ2**	883.160	-
**df**	265	-
**X** ^**2**^ **/df**	3.333	< 5
**P-value**	0.000	P > 0.05
**CFI**	0.901	> 0.9
**GFI**	0.927	> 0.9
**RMSEA**	0.050	< 0.08
**IFI**	0.901	> 0.9
**PNFI**	0.763	> 0.5
**PCFI**	0.795	> 0.5
**PGFI**	0.756	> 0.5
**AGFI**	0.910	> 0.8

**Table 3:** Factor loadings of the Persian version of long form of LOSS

Subscales	Items	Factor loadings
**F1: Causes/triggers**	1. If you asked someone directly ‘Do you feel like killing yourself?’ it will likely lead that person to make a suicide attempt (F)	0.406
	2. Those who attempt suicide do so only to manipulate others and attract attention to themselves (F)	0.577
	3. Very few people have thoughts about suicide (F)	0.565
	4. If assessed by a psychiatrist, everyone who suicides would be diagnosed as depressed (F)	0.591
	5. A suicidal person will always be suicidal and entertain thoughts of suicide (F)	0.623
	6. Talking about suicide always increases the risk of suicide (F)	0.522
	7. Motives and causes of suicide are readily and easily established (F)	0.515
	8. Media coverage of suicide will inevitably encourage other people to attempt suicide (F)	0.485
	9. Most people who attempt suicide fail to kill themselves (T)	0.527
	10. *A person who suicides is mentally ill (F)*	*Deleted*
**F2: Risk factors**	11. Most people who suicide are psychotic (F)	0.558
	12. People with relationship problems or financial problems have a higher risk of suicide (T)	0.512
	13. A person who has made a past suicide attempt is more likely to attempt suicide again than someone who has never attempted (T)	0.512
	14. Men are more likely to suicide than women (T)	0.497
	15. People who are anxious or agitated have a higher risk of suicide (T)	0.570
	16. There is a strong relationship between alcoholism and suicide (T)	0.513
	17. Most people who suicide are younger than 30 (F)	0.489
**F3: Signs and symptoms**	18. Not all people who attempt suicide plan their attempt in advance (T)	0.614
	19. People who talk about suicide rarely kill themselves (F)	0.610
	20. People who want to attempt suicide can change their mind quickly (T)	0.591
	21. Most people who suicide don’t make future plans (F)	0.539
	22. A time of high suicide risk in depression is at the time when the person begins to improve (T)	0.581
**F4: Treatment/Prevention**	23. Nothing can be done to stop people from making the attempt once they have made up their minds to kill themselves (F)	0.709
	24. Only experts can help people who want to suicide (F)	0.616
	25. People who have thoughts about suicide should not tell others about it (F)	0.681
	26. Seeing a psychiatrist or psychologist can help prevent someone from suicide (T)	0.556

*F = False, T = True

### Reliability assessment

For all questions of LOSS (25 items), the Cronbach’s alpha coefficient was 0.859 and McDonald omega coefficient was 0.866. In test-retest, for all questions ICC was 0.895. Reliability results for causes/triggers, risk factors, signs/symptoms, and treatment/prevention are mentioned in Table 4. Based on the results of Table 5, there was a significant positive correlation between all subscales (p < 0.001) (Table 5).

**Table 4:** Descriptive statistics of the Persian version of long form of LOSS

Subscales	Item	Range	Cronbach’s alpha coefficients	McDonald’s omega coefficients	Intraclass Correlation Coefficient (ICC)	95% Confidence Interval		P-value
						Lower Bound	Upper Bound	
**Factor 1: Causes/triggers**	9	0–9	0.624	0.640	0.885	0.761	0.945	< 0.001
**Factor 2: Risk factors**	7	0–7	0.792	0.799	0.900	0.793	0.952	< 0.001
**Factor 3: Signs and symptoms**	5	0–5	0.706	0.722	0.925	0.840	0.964	< 0.001
**Factor 4: Treatment/Prevention**	4	0–4	0.640	0.684	0.906	0.803	0.955	< 0.001
**Total LOSS**	25	0–25	0.859	0.866	0.895	0.781	0.950	< 0.001

**Table 5:** Pearson correlation between subscales of long form of LOSS

Subscales		Causes/triggers	Risk factors	Signs and symptoms
**Causes/triggers**	Pearson Correlation	1		
	Sig. (2-tailed)			
**Risk factors**	Pearson Correlation	0.583**	1	
	Sig. (2-tailed)	0.000		
**Signs and symptoms**	Pearson Correlation	0.600**	0.579**	1
	Sig. (2-tailed)	0.000	0.000	
**Treatment/Prevention**	Pearson Correlation	0.470**	0.476**	0.569**
	Sig. (2-tailed)	0.000	0.000	0.000
**. Correlation is significant at the 0.01 level (2-tailed).				

## Discussion

Based on a literature review, no psychometric studies of LOSS have been investigated in Iranian populations. The original LOSS questionnaire consisted of 26 questions, in this study, 1 item was removed after evaluation, and the modified Persian version was confirmed as 25 items and 4 factors. Based on the results, the Persian version appears to be useful for measuring LOSS for age groups of different literacy levels in the community.

Previous studies suggest that the McDonald’s omega coefficients provide a more accurate approximation of scale reliability and is a more reasonable indicator than internal compatibility than Cronbach’s alpha. Therefore, when creating a new criterion, the reliability coefficient above 0.70 is considered acceptable [36, 37]. In this study, the Omega McDonald coefficient and Cronbach’s alpha coefficient were used to measuring the reliability of the instrument, which were appropriated values 0.866 for McDonald’s omega coefficients and 0.859 for Cronbach’s alpha. Also, the ICC rate for all questions is 0.895, which is acceptable. Although there is no similar study examining the psychometric characteristics of LOSS in Iran, but in Rafati study[38], only the psychometric characteristics of another questionnaire designed to examine social attitudes to suicide were examined. In Rafati study, the Persian version of the Social Attitudes to Suicide Questionnaire was validated and Cronbach’s alpha (0.94), McDonald’s omega coefficients (0.943) and ICC index (0.998) were acceptable [38].

In our study and in the CFA stage, one question was deleted from the original version and the final version of the Persian LOSS was confirmed with 25 questions and four factors of causes/ triggers (9 questions), risk factors (7 questions), sign/symptoms (5 questions), and treatment/ prevention (4 questions). One of the reasons for deleting item S10 (“A person who suicides is mentally ill”) may be that the concept of this issue is not easily understood by the Iranian public unless. On the other hand, the concept and meaning of item S10 is somewhat similar to item 11, and it can even be said that item S11 is easier for Iranian people to understand than item S10.

In a study, the Malaysian version of the LOSS with the Rasch model was validated with a one-dimensional scale and 26 items [18]. The difference in dimensionality can be attributed to the model used, since one of the assumptions of the model is that it is one-dimensional. As can be seen in our study, all the dimensions of the main questionnaire were confirmed, although one question was removed from the final version.

According to the results of the Öztürk study, the Turkish version of LOSS based on Item Response Theory showed a single subscale with an ICC calculation of 0.87 [23]. Therefore, the observed difference is somewhat justified and due to the differences between the models used. In the Chan study in the Australian medical students, Cronbach’s alphabet was reported 0.71 for LOSS questionnaire [20].

Studies that investigated the process of suicide and its death over five years in southern Iran showed that the suicide process had increased during the period under investigation in the general population [39] as well as adolescents [40], and the elderly [41]. Therefore, given the importance of the suicide and the use of this tool in other countries [18, 20, 23], this valid and reliable instrument can be used to determine the status of suicide literacy of the Iranian population and take necessary preventive programs if needed.

### Strengths and limitation

Limitations of this study include changes and reductions in the number of questions of the modified Persian version of the questionnaire compared to the original version, resulting in changes in the questionnaire scores. One of the strengths of this study is the use of a high sample size and examination in public population from different age and social groups. Another limitation of this study is that school-age students (15 to 18 years old) were not included in the study, therefore, this may reduce the external validity of the study.

## Conclusion

The Persian long version of LOSS with four subscales and 25 items is a valid and reliable instrument. Therefore, given the number of appropriate questions and ease of use, it will be used to investigate the status of suicide literacy in different populations and different groups and finally, help health decision makers design and implement appropriate intervention programs if necessary.

## Electronic supplementary material

Below is the link to the electronic supplementary material.

Supplementary Material 1

## Data Availability

All data generated or analysed during this study are included in this published article.

## Abbreviations

**LOSS**: Literacy of Suicide Scale
**CFA**: Confirmatory factor analysis
**F1**: Causes/triggers
**F2**: Risk factors
**F3**: Signs and symptoms
**F4**: Treatment/Prevention
**PCFI**: parsimony comparative fit index
**AGFI**: Adjusted goodness of fit index
**GFI**: Goodness of fit index
**IFI**: Incremental fit index
**RMSEA**: Square error of approximation
**PNFI**: Parsimonious normed fit index
**PGFI**: Parsimony goodness-of-fit index
**x2/df**: Chi-square ratio to degree of freedom
**CFI**: Comparative fit index

## References

[1] Soomro GM, Kakhi S. Deliberate self-harm (and attempted suicide). *BMJ clinical evidence* 2015, 2015. PMC4451502 26032238

[2] Bursztein Lipsicas C, Mäkinen IH, Apter A, De Leo D, Kerkhof A, Lönnqvist J, Michel K, Salander Renberg E, Sayil I, Schmidtke A (2012). Attempted suicide among immigrants in european countries: an international perspective. Soc Psychiatry Psychiatr Epidemiol.

[3] Artenie AA, Bruneau J, Zang G, Lespérance F, Renaud J, Tremblay J, Jutras-Aswad D (2015). Associations of substance use patterns with attempted suicide among persons who inject drugs: can distinct use patterns play a role?. Drug Alcohol Depend.

[4] Karimi A, Bazyar J, Malekyan L, Daliri S (2022). Prevalence of suicidal ideation and suicide attempts after disaster and mass casualty incidents in the world: a systematic review and meta-analysis. Iran J psychiatry.

[5] Crispim MO, Santos C, Frazão IDS, Frazão C, Albuquerque RCR, Perrelli JGA (2021). Prevalence of suicidal behavior in young university students: a systematic review with meta-analysis. Rev Latinoam Enferm.

[6] Balvardi M, Imani-Goghary Z, Babaee K, Izadabadi Z. Suicide and attempted suicide epidemiology in sirjan in 2018.International journal of high risk behaviors and addiction2021, 10(2).

[7] Sun X, Shi Y, Zeng Q, Wang Y, Du W, Wei N, Xie R, Chang C (2013). Determinants of health literacy and health behavior regarding infectious respiratory diseases: a pathway model. BMC Public Health.

[8] Visscher BB, Steunenberg B, Heijmans M, Hofstede JM, Devillé W, van der Heide I, Rademakers J (2018). Evidence on the effectiveness of health literacy interventions in the EU: a systematic review. BMC Public Health.

[9] Batterham PJ, Calear AL, Christensen H (2013). Correlates of suicide stigma and suicide literacy in the community. Suicide and Life-Threatening Behavior.

[10] Fisher D (2005). The literacy educator’s role in suicide prevention. J Adolesc Adult Lit.

[11] Sharaf AY, Ossman LH, Lachine OA (2012). A cross-sectional study of the relationships between illness insight, internalized stigma, and suicide risk in individuals with schizophrenia. Int J Nurs Stud.

[12] Calear AL, Batterham PJ, Christensen H (2014). Predictors of help-seeking for suicidal ideation in the community: risks and opportunities for public suicide prevention campaigns. Psychiatry Res.

[13] Al-Shannaq Y, Aldalaykeh M. Suicide literacy, suicide stigma, and psychological help seeking attitudes among Arab youth.Current psychology2021:1–13. 10.1007/s12144-021-02007-9 PMC8214717 34177209

[14] Ludwig J, Dreier M, Liebherz S, Härter M, von dem Knesebeck O (2022). Suicide literacy and suicide stigma–results of a population survey from Germany. J mental health.

[15] Batterham PJ, Han J, Calear AL, Anderson J, Christensen H (2019). Suicide stigma and suicide literacy in a clinical sample. Suicide and Life-Threatening Behavior.

[16] Aldalaykeh M, Dalky H, Shahrour G, Rababa M (2020). Psychometric properties of two arabic suicide Scales: Stigma and literacy. Heliyon.

[17] Ferlatte O, Salway T, Oliffe JL, Rice SM, Gilbert M, Young I, McDaid L, Ogrodniczuk JS, Knight R (2021). Depression and suicide literacy among canadian sexual and gender minorities. Archives of suicide research.

[18] Phoa PKA, Razak AA, Kuay HS, Ghazali AK, Rahman AA, Husain M, Bakar RS, Gani FA. The Malay Literacy of Suicide Scale: a Rasch model validation and its correlation with mental health literacy among Malaysian parents, caregivers and teachers. Healthcare: 2022:MDPI; 2022:p. 1304. 10.3390/healthcare10071304 PMC9317984 35885830

[19] Arafat SY, Hussain F, Hossain MF, Islam MA, Menon V (2022). Literacy and stigma of suicide in Bangladesh: Scales validation and status assessment among university students. Brain and behavior.

[20] Chan WI, Batterham P, Christensen H, Galletly C (2014). Suicide literacy, suicide stigma and help-seeking intentions in australian medical students. Australasian Psychiatry.

[21] Han J, Batterham PJ, Calear AL, Wu Y, Shou Y, Van Spijker BA (2017). Translation and validation of the chinese versions of the suicidal ideation attributes scale, stigma of suicide scale, and literacy of suicide scale. Death Stud.

[22] Ibrahim S. Suicide literacy and Laypersons’ ability to accurately recognize suicide warning signs and risk factors. Illinois State University; 2019.

[23] Öztürk A, Akın S. The turkish version of literacy of suicide scale (LOSS): validity and reliability on a sample of turkish university students. 2016.

[24] Tabatchnick BG, Fidell LS. Using multivariate statistics. *Needham Heights, MA* 2001.

[25] Williams B, Onsman A, Brown T. Exploratory factor analysis: A five-step guide for novices.Australasian journal of paramedicine2010, 8(3).

[26] Calear AL, Batterham PJ, Trias A, Christensen H. The Literacy of Suicide Scale: Development, validation, and application.Crisis: The Journal of Crisis Intervention and Suicide Prevention2021.

[27] Organization WH. Process of translation and adaptation of instruments. 2009. In.; 2010.

[28] Taghizadeh Z, Ebadi A, Montazeri A, Shahvari Z, Tavousi M, Bagherzadeh R (2017). Psychometric properties of health related measures. Part 1: translation, development, and content and face validity. Payesh.

[29] Henry JW, Stone RW (1994). A structural equation model of end-user satisfaction with a computer-based medical information system. Inform Resour Manage J (IRMJ).

[30] Lomax RG, Schumacker RE. A beginner’s guide to structural equation modeling. psychology press; 2004.

[31] Kline R (2005). Details of path analysis. Principles and practice of structural equation modeling. In.

[32] Schreiber JB, Nora A, Stage FK, Barlow EA, King J (2006). Reporting structural equation modeling and confirmatory factor analysis results: a review. J educational Res.

[33] Nunnally JC. Psychometric theory 3E. Tata McGraw-Hill Education; 1994.

[34] Bland JM, Altman DG (1997). Statistics notes: Cronbach’s alpha. BMJ.

[35] Koo TK, Li MY (2016). A Guideline of selecting and reporting Intraclass correlation coefficients for Reliability Research. J Chiropr Med.

[36] Revelle W, Zinbarg RE (2009). Coefficients alpha, beta, omega, and the glb: comments on Sijtsma. Psychometrika.

[37] Viladrich C, Angulo-Brunet A, Doval E (2017). A journey around alpha and omega to estimate internal consistency reliability. Annals of psychology.

[38] Rafati A, Janani L, Malakouti SK, Motevalian SA, Kabiri A, Pasebani Y, Shalbafan M. Evaluation of Psychometric Properties of the Persian Version of the Predicaments Questionnaire, Exploring Social Attitudes to Suicide. 2022. 10.3389/fpubh.2022.1061673 PMC9872659 36703832

[39] Mirahmadizadeh A, Rezaei F, Mokhtari AM, Gholamzadeh S, Baseri A (2020). Epidemiology of suicide attempts and deaths: a population-based study in Fars, Iran (2011–16). J Public Health.

[40] Mokhtari AM, Gholamzadeh S, Salari A, Hassanipour S, Mirahmadizadeh A (2019). Epidemiology of suicide in 10–19 years old in southern Iran, 2011–2016: a population-based study on 6720 cases. J Forensic Leg Med.

[41] Mokhtari AM, Sahraian S, Hassanipour S, Baseri A, Mirahmadizadeh A (2019). The epidemiology of suicide in the elderly population in Southern Iran, 2011–2016. Asian J psychiatry.

