# Supplementary material for: Title page: psychometric properties of literacy of suicide scale (LOSS) in iranian population: long form
Source: BMC Public Health. 2023 Mar 30;23:608. doi: 10.1186/s12889-023-15528-8 (PMC10064757; doi:10.1186/s12889-023-15528-8)
Supplement: Supplementary file 1 — Supplementary Material 1 [file 12889_2023_15528_MOESM1_ESM.docx]

**Additional file 1**

**Table S1:** The final version of Literacy of Suicide Scale (LOSS) with 25 items and four factors

| **Subscales** | **Items** |
| --- | --- |
| **F1: Causes/triggers** | 1. If you asked someone directly ‘Do you feel like killing yourself?’ it will likely lead that person to make a suicide attempt (F) |
|  | 1. Those who attempt suicide do so only to manipulate others and attract attention to themselves (F) |
|  | 1. Very few people have thoughts about suicide (F) |
|  | 1. If assessed by a psychiatrist, everyone who suicides would be diagnosed as depressed (F) |
|  | 1. A suicidal person will always be suicidal and entertain thoughts of suicide (F) |
|  | 1. Talking about suicide always increases the risk of suicide (F) |
|  | 1. Motives and causes of suicide are readily and easily established (F) |
|  | 1. Media coverage of suicide will inevitably encourage other people to attempt suicide (F) |
|  | 1. Most people who attempt suicide fail to kill themselves (T) |
| **F2: Risk factors** | 1. Most people who suicide are psychotic (F) |
|  | 1. People with relationship problems or financial problems have a higher risk of suicide (T) |
|  | 1. A person who has made a past suicide attempt is more likely to attempt suicide again than someone who has never attempted (T) |
|  | 1. Men are more likely to suicide than women (T) |
|  | 1. People who are anxious or agitated have a higher risk of suicide (T) |
|  | 1. There is a strong relationship between alcoholism and suicide (T) |
|  | 1. Most people who suicide are younger than 30 (F) |
| **F3: Signs and symptoms** | 1. Not all people who attempt suicide plan their attempt in advance (T) |
|  | 1. People who talk about suicide rarely kill themselves (F) |
|  | 1. People who want to attempt suicide can change their mind quickly (T) |
|  | 1. Most people who suicide don’t make future plans (F) |
|  | 1. A time of high suicide risk in depression is at the time when the person begins to improve (T) |
| **F4: Treatment/Prevention** | 1. Nothing can be done to stop people from making the attempt once they have made up their minds to kill themselves (F) |
|  | 1. Only experts can help people who want to suicide (F) |
|  | 1. People who have thoughts about suicide should not tell others about it (F) |
|  | 1. Seeing a psychiatrist or psychologist can help prevent someone from suicide (T) |
